# Potential correlation of allograft infiltrating group 2 innate lymphoid cells with acute rejection after liver transplantation

**DOI:** 10.3389/fimmu.2022.953240

**Published:** 2022-07-28

**Authors:** Jie Sun, Guang-Peng Zhou, Shi-Peng Li, Xiao-Jie Chen, Jin-Ming Zhang, Yi-Zhou Jiang, Bin Cui, Hai-Ming Zhang, Li-Ying Sun, Zhi-Jun Zhu

**Affiliations:** ^1^ Liver Transplantation Center, National Clinical Research Center for Digestive Diseases, Beijing Friendship Hospital, Capital Medical University, Beijing, China; ^2^ Clinical Research Center for Pediatric Liver Transplantation, Capital Medical University, Beijing, China; ^3^ Department of Critical Liver Disease, Liver Research Center, Beijing Friendship Hospital, Capital Medical University, Beijing, China

**Keywords:** ILC2, Treg cell, immune tolerance, hepatic immune microenvironment, spatial distribution

## Abstract

Accumulating evidence indicates the critical roles of group 2 innate lymphoid cells (ILC2s) in immunoregulation. However, the role of ILC2s in acute rejection after liver transplantation (LT) remains elusive. In this study, we analyzed the frequency, counts, and signature cytokines of ILC2s in liver transplant recipients by flow cytometric analysis and multiplex immunofluorescence assay. We also assessed the spatial distribution and correlation between hepatic ILC2s and Treg cells. The changes of ILC2s were dynamically monitored in the mouse LT model. We found that the frequencies of circulating ILC2s were comparable in liver transplant recipients with either rejection or non-rejection compared with the control group. The hepatic ILC2s counts were significantly increased in the rejection group than in the non-rejection and control groups, and a similar trend was observed for Treg cells. In the mouse LT model, allograft infiltrating ILC2s dramatically increased within 14 days post-transplant. The frequency of ILC2s in bone marrow significantly increased at 7 days post-transplant and rapidly decreased at 14 days after LT. Similarly, there was a significant increase in the frequency of splenic ILC2s within two weeks post-transplant. Multiplex immunofluorescence assay showed a close correlation between hepatic ILC2s and Treg cells by analyzing their spatial distribution and distance. In conclusion, the number of allograft infiltrating ILC2s was closely related to rejection after LT. Allograft infiltrating ILC2s may play inhibitory roles in posttransplant immune homeostasis, favoring resolution of liver allograft rejection by interacting with Treg cells or promoting the migration of Tregs cells into the liver allograft.

## Introduction

Liver transplantation (LT) has been established as the only curative therapeutic option for patients with end-stage liver disease. However, allograft rejection contributing to significantly increased risk of graft failure, all-cause mortality, and graft failure-related death has compromised the short- and long-term prognosis of liver transplant recipients (1). There is mounting evidence that hepatic immune microenvironment, liver-resident and circulating immune cells, and specific molecular pathways are implicated in regulating the balance between transplant immune tolerance and rejection (2–4). It is well known that immunological rejection is triggered by recognizing allogeneic antigens by antigen-presenting cells (APCs). In the presence of appropriate co-stimulatory molecules and a pro-inflammatory cytokine milieu, activated APCs interact with naïve alloreactive T cells, resulting in the proliferation of alloreactive CD4+ and CD8+ effector T cells and subsequent B-cell proliferation (2, 4). Then circulating leukocytes, including activated effector T cells, are recruited into the liver allograft to mediate liver damage (5).

Innate lymphoid cells (ILCs), characterized by lacking adaptive antigen-specific receptors, have been newly identified as a heterogeneous subpopulation of innate immune cells, which are classified into three groups, group 1 ILCs (ILC1s), group 2 ILCs (ILC2s), and group 3 ILCs (ILC3s), based on their expression of transcription factors, phenotypic markers, and effector cytokines (6, 7). ILC2s are dependent on RORα and GATA3 and produce the type 2 T helper (Th2) cell-associated cytokines, such as interleukin (IL)-4, IL-5, IL-9, and IL-13, in response to stimulation with the cytokines IL-25, IL-33 and thymic stromal lymphopoietin (8, 9). Although ILC2s have previously been studied mainly in the field of inflammatory and allergic diseases (10, 11), several recently published studies have demonstrated their substantial roles in various liver disorders, such as acute liver injury, chronic hepatitis, hepatic fibrosis, and hepatocellular carcinoma (12–15). Recently, Gomez-Massa et al. found that the number of circulating ILC2s and their signature cytokine productions were comparable in liver transplant recipients during the early peri-transplant period compared to control subjects (16). Nevertheless, given the tissue-resident properties of ILC2s, the distribution of the ILC2s in the liver allograft may be more relevant in driving the immune balance toward either a pro-inflammatory state (rejection) or an anti-inflammatory condition (tolerance) (16). In addition, previous studies have described that ILC2s might participate in transplant immunology (17, 18). A recently published study by Huang et al. directly demonstrated that IL-10-producing ILC2s could provide the maximal suppressive effect and graft protection within the islet allograft (19). However, currently available data about their function in liver allografts are still scarce, and thus whether ILC2s are involved in alloreactive responses following LT remains unclear.

Therefore, our study aims to explore the alterations in the frequency, counts, and functional cytokines of ILC2s in liver transplanted patients and mouse orthotopic liver transplant model. We found that the counts of allograft infiltrating ILC2s were dramatically increased after LT, especially in those experiencing acute rejection, and similar results were observed for CD4+CD25+ regulatory T (Treg) cells. Multiplex immunofluorescence assay showed a close correlation between allograft infiltrating ILC2s and Treg cells by analyzing their spatial distribution and distance. Collectively, we postulate that allograft infiltrating ILC2s may play an inhibitory role in posttransplant immune homeostasis, favoring resolution of liver allograft rejection by interacting with Treg cells or promoting the migration of Tregs cells into the liver allograft.

## Results

### Frequencies of circulating ILC2s were comparable in liver transplant recipients with either rejection or non-rejection compared with the control group

**Figure 1:**
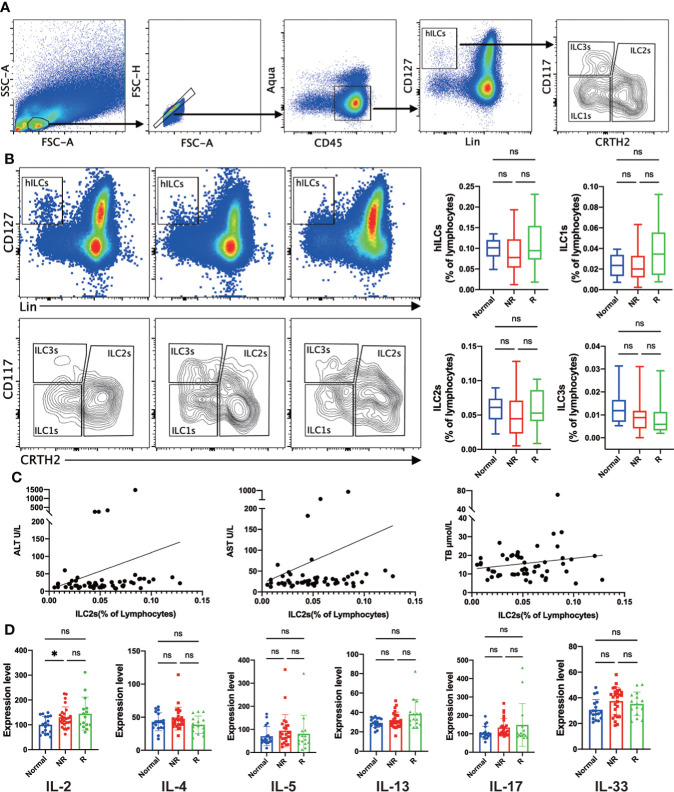
Similar frequencies of circulating helper innate lymphoid cells (hILCs), group 1 Innate lymphoid cells (ILC1s), group 2 Innate lymphoid cells (ILC2s), and group 3 Innate lymphoid cells (ILC3s) were detected in liver transplant recipients with either rejection or non-rejection compared with the control group. **(A)** The gating strategy to identify human hILC, ILC1s, ILC2s, and ILC3s in peripheral blood. **(B)** Representative flow cytometric analysis showed a similar frequency of circulating hILCs, ILC1s, ILC2s, and ILC3s were detected liver transplant recipients with either rejection or non-rejection compared with the control group. **(C)** Correlation analysis showed no correlations between the frequency of circulating ILC2s and the plasma levels of ALT, AST, and TB. **(D)** The plasma level of IL-2 was significantly increased in the non-rejection group compared with the control group, and there was no significant difference in the plasma level of IL-2, IL-4, IL-5, IL-13, IL-17, and IL-33 in any other comparison between the two groups. *p < 0.05; **p < 0.01; ***p < 0.001. ****p < 0.0001, ns *p > 0.05*.

To investigate the role of helper ILCs (hILCs) in the acute rejection after LT, we performed a comparative analysis of hILCs (CD45+Lin-CD127+) and the distinct subgroups, including ILC1s (CD45+Lin-CD127+CRTH2-CD117-), ILC2s (CD45+Lin-CD127+ CRTH2+), and ILC3s (CD45+Lin-CD127+ CRTH2-CD117+) in the peripheral blood of liver transplant recipients and healthy controls (**Figure 1A**). Results showed that the frequencies of hILCs was comparable in liver transplant recipients with either rejection or non-rejection compared with the control group (**Figure 1B**). Similar frequencies of circulating ILC1s, ILC2s, and ILC3s were observed in liver transplant recipients with either rejection or non-rejection compared with healthy controls (**Figure 1B**). Furthermore, no correlations were found between the frequencies of circulating ILC2s and the levels of alanine aminotransferase (ALT), aspartate aminotransferase (AST), and total bilirubin (TB) (**Figure 1C**).

ILC2s are characterized for their immune regulation ability by plenty of cytokines, so we evaluated the plasma levels of signature cytokines by enzyme-linked immunosorbent assay (ELISA) to assess the function of ILC2s. IL-33 was measured as a potent ILC2s stimulator, and IL-4, IL-5, and IL-13 were the most representative effector of ILC2s. Besides, IL-2 and IL-17 were also analyzed as typical pro-inflammation signals. Results showed that the plasma level of IL-2 was significantly increased in non-rejection group compared with the control group, and there was no significant difference in the plasma level of IL-2, IL-4, IL-5, IL-13, IL-17, and IL-33 in any other comparison between the two groups (**Figure 1D**).

### Allograft infiltrating ILC2s increased after liver transplantation

**Figure 2:**
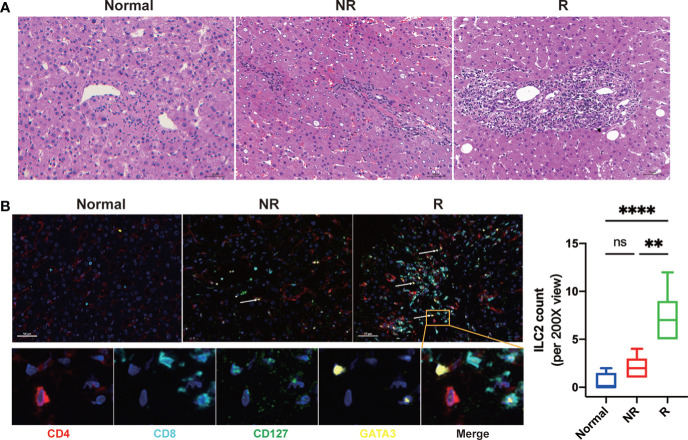
Significantly increased allograft infiltrating immune cells and group 2 Innate lymphoid cells (ILC2s) were related to acute rejection after liver transplantation. **(A)** Representative images of hematoxylin and eosin (H&E) of fixed and paraffin-embedded human liver tissue sections indicated that compared with that of the normal and non-rejection group, the infiltrations of immune cells in allograft were significantly increased in the rejection group. Scale bar, 50 μm. **(B)** Multiplex immunofluorescent staining of ILC2s in liver tissues of each group showed that hepatic ILC2s (CD127+GATA3+; white arrow) were increased after LT, particularly in the rejection group. Scale bar, 50 μm. Fixed and paraffin-embedded tissue sections were labeled against CD4 (red), CD8 (cyan), CD127 (green), and GATA3 (yellow) using Opal reagents (Akoya Biosciences). Whole slides were scanned using the Vectra Polaris multispectral imaging platform (Akoya Biosciences), and image analysis was performed using the InForm 2.4.8 Image Analysis Software (Akoya Biosciences). *p < 0.05; **p < 0.01; ***p < 0.001. ****p < 0.0001, ns *p > 0.05*.

Immunohistochemical staining of liver tissues showed that many immune cells infiltrated the portal regions and hepatic lobular compared with healthy controls after LT (**Figure 2A**). To access the distribution of ILC2s in the liver allograft, we performed the multiplex immunofluorescence assay for CD4, CD8, CD127, GATA3, and DAPI. The result showed that hepatic ILC2s (CD4-CD8-CD127+GATA3+) were barely seen in the healthy controls but accumulated in the portal regions and hepatic lobular after LT, particularly in the rejection group (**Figure 2B**).

### ILC2s increased in the bone marrow and successively migrated to liver allograft after liver transplantation

**Figure 3:**
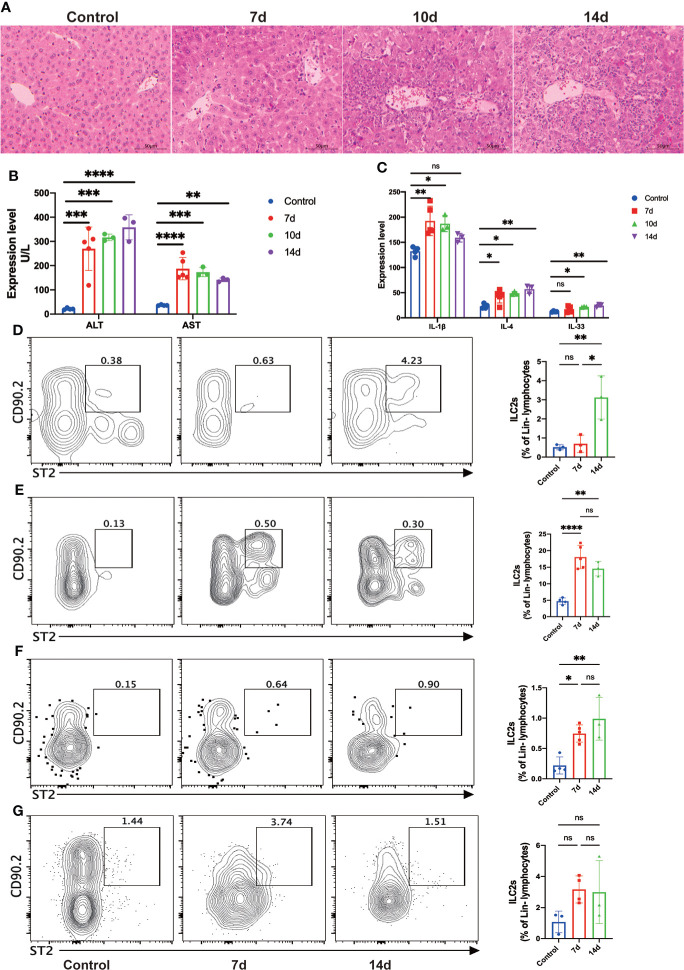
Allograft infiltrating group 2 Innate lymphoid cells (ILC2s) were significantly increased after liver transplantation (LT) in the mouse LT model. **(A)** Representative hematoxylin and eosin (H&E) staining of fixed and paraffin-embedded mouse liver tissues after LT. Scale bar, 50 μm. **(B)** The plasma levels of ALT and AST were significantly increased after LT (control group, *n* = 4; 7d group, *n* = 5; 10d group, *n* = 3; 14d group, *n* = 3). **(C)** The plasma levels of IL-1β, IL-4, and IL-33 were increased after LT (control group, *n* = 4; 7d group, *n* = 5; 10d group, *n* = 3; 14d group, *n* = 3). **(D)** Hepatic ILC2s dramatically expanded at 14 days after LT (control group, *n* = 3; 7d group, *n* = 3; 14d group, *n* = 3). **(E)** The frequency of ILC2s in bone marrow was significantly increased at seven days post-transplant and slightly decreased after 14 days (control group, *n* = 4; 7d group, *n* = 5; 14d group, *n* = 3). **(F)** There was a significant increase in the frequency of splenic ILC2s within two weeks post-transplant (control group, *n* = 4; 7d group, *n* = 5; 14d group, *n* = 3). **(G)** No considerable increase of ILC2s was observed in mesenteric lymph nodes (control group, *n* = 3; 7d group, *n* = 4; 14d group, *n* = 3). *p < 0.05; **p < 0.01; ***p < 0.001. ****p < 0.0001, ns *p > 0.05*.

To further demonstrate the findings in human, we established the mouse orthotopic liver transplant model and dynamically accessed the changes of ILC2s in different tissues. Hematoxylin and eosin (H&E) staining showed that hepatocytes and bile duct injury were generally exacerbated after LT (**Figure 3A**). On postoperative day (POD) 7, liver grafts presented a relatively conserved locular structure, although considerable degeneration and cholestasis had been visible. On POD 10, the necrotic liver injury almost occupied the entire periportal regions, and immune cell infiltration was accumulated around the portal areas and sinusoids. There was no obvious tissue repairment or further damage on POD 14 (**Figure 3A**). The plasma levels of ALT and AST, in accordance with the hepatic histology, were significantly increased after LT (**Figure 3B**). We analyzed relevant cytokines expression in plasm and found pro-inflammation cytokine IL-1β increased rapidly after surgery (**Figure 3C**). It has been reported that the injured hepatocytes can secrete IL-33 (the activator and effector molecular of ILC2s) to induce an immune response. Results showed that IL-33 and IL-4 significantly increased compared with the healthy control, which may indicate the activation of ILC2s (**Figure 3C**). The above results suggested that the mouse orthotopic LT model exhibited severe acute rejection within the first 14 days.

Then we performed flow cytometry to investigate the accurate changes of ILC2s within the different tissues. Hepatic ILC2s barely exist under normal conditions and early period after surgery, while dramatically expanded at 14 days after LT (**Figure 3D**). Bone marrow (BM) is the proliferation and differentiation position of hILCs, including ILC2s. The frequencies of ILC2s in the BM increased rapidly in the early post- LT stage and gradually decreased at 14 days after LT (**Figure 3E**). In the meantime, we investigated the constitution of spleen immune cells. Although hILCs didn’t accumulate within the spleen, ILC2s increased significantly after LT (**Figure 3F**). Mesenteric lymph nodes (mLN) showed the same trends as the spleen, although with no statistical difference (**Figure 3G**). Collectively, we assume that ILC2s will vastly increase during the early period after LT and gradually migrate to the allogeneic graft.

### Circulating Treg cells decreased, but hepatic Treg cells increased after liver transplantation

**Figure 4:**
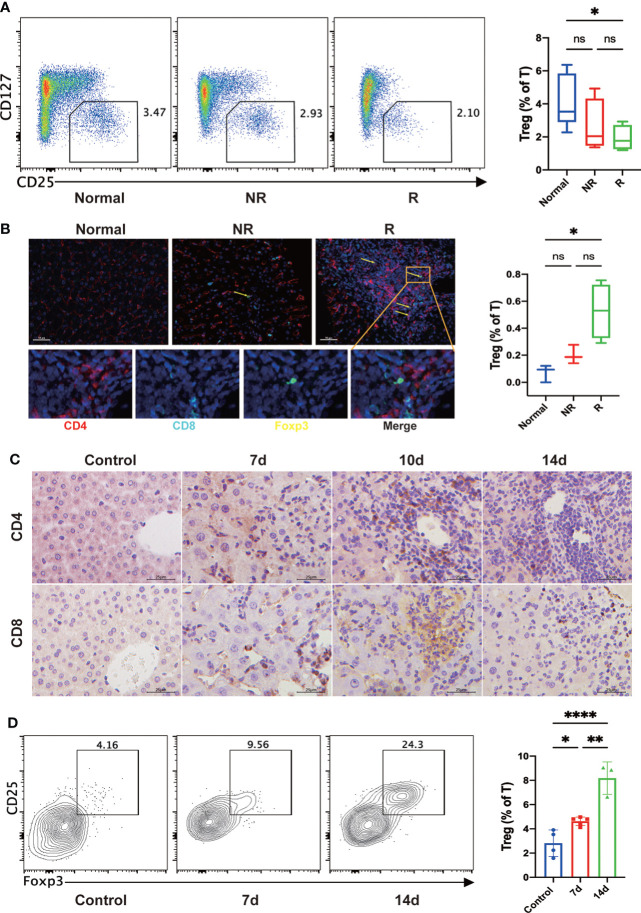
Circulating Treg cells were significantly decreased, and hepatic Treg cells were increased in the rejection group compared with the healthy controls. **(A)** The frequency of circulating Treg cells was decreased after liver transplantation (LT), particularly in the rejection group. **(B)** Multiplex immunofluorescent staining of Tregs in the fixed and paraffin-embedded human tissue sections of each group showed that hepatic Treg cells (CD4+Foxp3+; yellow arrow) were increased after LT, particularly in the rejection group. Scale bar, 50 μm. Fixed and paraffin-embedded tissue sections were labeled against CD4 (red), CD8 (cyan), and Foxp3 (yellow) using Opal reagents (Akoya Biosciences). Whole slides were scanned using the Vectra Polaris multispectral imaging platform (Akoya Biosciences), and image analysis was performed using the InForm 2.4.8 Image Analysis Software (Akoya Biosciences). **(C)** In the mouse LT model, immunohistochemistry staining of fixed and paraffin-embedded mouse liver tissues showed that CD4+ and CD8+ T cells gradually increased in the portal regions and hepatic sinusoids after LT. **(D)** In the mouse LT model, representative flow cytometric analysis showed that hepatic Treg cells were significantly increased after LT, especially two weeks later. *p < 0.05; **p < 0.01; ***p < 0.001. ****p < 0.0001, ns *p > 0.05*.

As Treg cells play a crucial role in the induction and maintenance of immune tolerance after LT, we then investigate the changes of Treg cells after LT. Results showed that the frequency of circulating Treg cells was significantly decreased in the rejection group than in the non-rejection and control groups (**Figure 4A**). In addition, multiplex immunofluorescence assay showed that hepatic Treg cells were significantly increased in liver transplant patients developing acute rejection compared with healthy controls (**Figure 4B**).

In the mouse LT model, immunohistochemical staining of liver tissues showed that CD4+ and CD8+ T cells gradually increased in the portal regions and hepatic sinusoids after LT (**Figure 4C**). Consistent with the results of human experiments, flow cytometry analysis showed that hepatic Treg cells were significantly increased after LT (**Figure 4D**).

### Allograft infiltrating ILC2s showed a close spatial correlation with Treg cells

**Figure 5:**
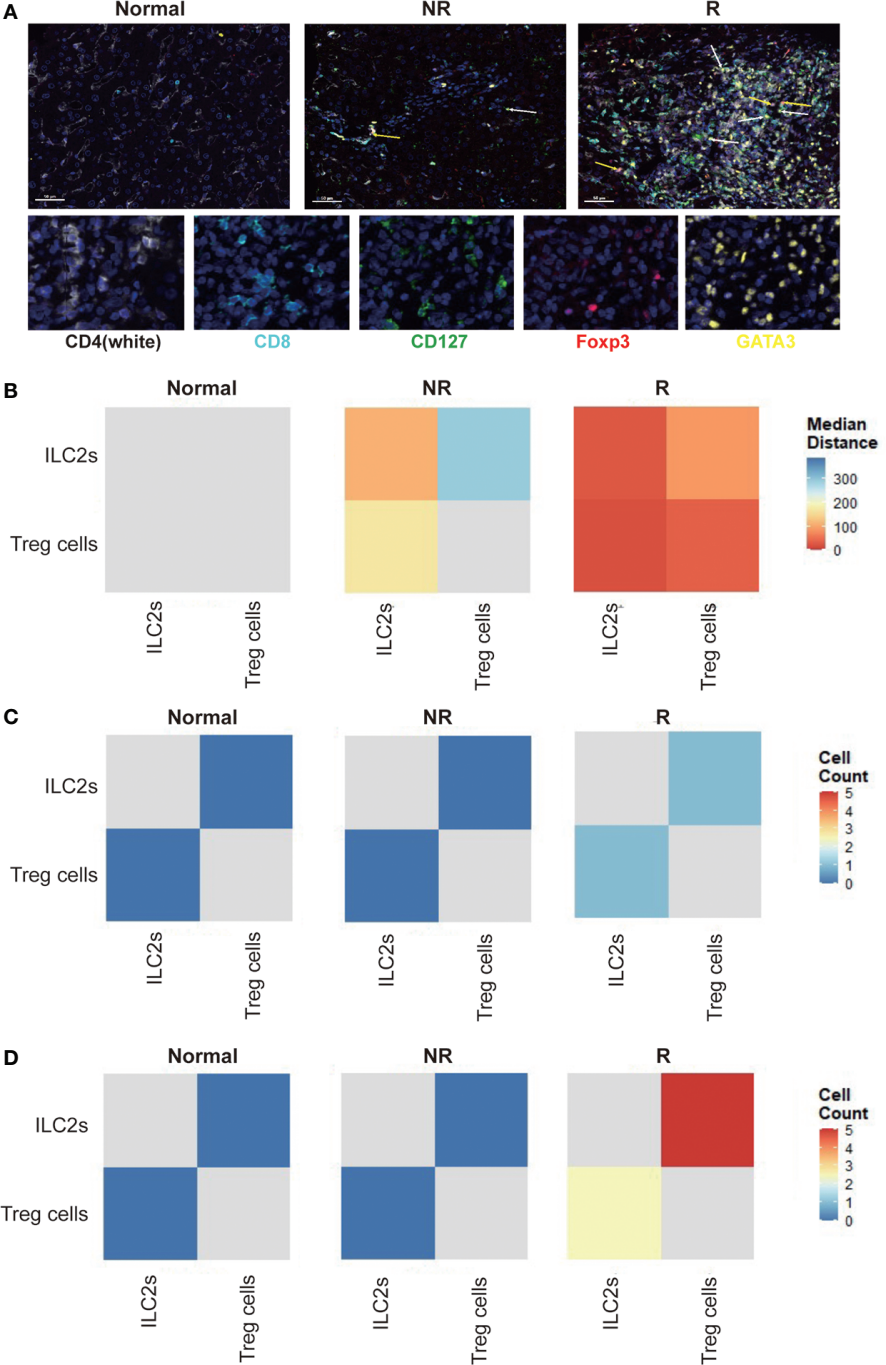
Allograft infiltrating group 2 Innate lymphoid cells (ILC2s) show a close spatial association with Treg cells. **(A)** Multiplex immunofluorescent staining of ILC2s (white arrow) and Treg cells (yellow arrow) in liver tissues of each group. Scale bar, 50 μm. Fixed and paraffin-embedded tissue sections were labeled against CD4 (white), CD8 (cyan), CD127 (green), Foxp3 (red), and GATA3 (yellow) using Opal reagents (Akoya Biosciences). Whole slides were scanned using the Vectra Polaris multispectral imaging platform (Akoya Biosciences), and image analysis was performed using the InForm 2.4.8 Image Analysis Software (Akoya Biosciences). Every individual ILC2 (CD127+GATA3+; white arrow) and Treg cells (CD4+Foxp3+; yellow arrow) in the liver section were identified based on their labeling. **(B)** Spatial distance between ILC2s and Treg cells in human liver sections. Every individual ILC2 or Treg cell in the liver section were identified based on their labeling (CD127+GATA3+ for ILC2s; CD4+Foxp3+ for Treg cells), and then the spatial distribution of ILC2 and Treg cells was determined. The distance between ILC2 and the nearest Treg cell and Treg cell and the nearest ILC2 was measured, and then the median distance between ILC2 and Treg cell was calculated. We drew heatmaps through the R software to exhibit the results. The X-axis and y-axis both represent ILC2s and Treg cells. The depth of the color indicates the spatial distance between ILC2s and Treg cells. The blue color indicates far distance and the red color close distance. Compared with healthy controls, the median distance between ILC2s and Treg cells was much closer in liver transplant recipients, particularly in the rejection group. **(C)** The number of Treg cells or ILC2s within a circle with a diameter of 15 microns centered on ILC2s and Treg cells were counted. The heatmaps were used to exhibit the results, and the x-axis and y-axis both represent ILC2s and Treg cells. The depth of the color indicates the cell counts of ILC2s and Treg cells. Blue color indicates less number and red color more number. Compared with healthy and non-rejection groups, there were more Treg cells around ILC2s within the range of 15 microns in the rejection group and more ILC2s around Treg cells. **(D)** Compared with healthy and non-rejection groups, there were more Treg cells around ILC2s within the range of 25 microns in the rejection group and more ILC2s around Treg cells.

To clarify the role of ILC2s in the immune microenvironment after LT, we applied multiplex immunofluorescent assays to analyze the spatial distribution of liver allograft infiltrating ILC2s and Treg cells, as well as their spatial distance (**Figure 5A**). We found that compared with healthy controls, the median distance between ILC2s and Treg cells was much closer in liver transplant recipients, particularly in the rejection group (**Figure 5B**). In addition, we found that compared with healthy controls and non-rejection group, there were more Treg cells around ILC2s within the range of 15 microns in the rejection group and more ILC2s around Treg cells (**Figure 5C**). Similar results were observed within the scope of 25 microns (**Figure 5D**). Thus, we presume that ILC2s might induce immune tolerance by migrating into the liver allograft and interacting with hepatic Treg cells or recruiting Treg cells to migrate into the liver allograft.

## Discussion

There is accumulating evidence suggesting that ILC2s play essential roles in regulating innate and adaptive immune responses, mainly in acute and chronic inflammatory diseases (20, 21). However, the potential immunoregulatory function of ILC2s in allograft rejection after LT has not been addressed. A previous study found that ILC2s counts in the peripheral blood and ILC2s-related cytokine levels were comparable between liver transplant recipients and normal populations (16). Notably, however, authors only analyzed the numbers and functions of ILC2s in the peripheral blood during the early peri-transplant period (14 days after LT) and did not investigate the ILC2s counts in the liver allograft, especially considering the tissue infiltration properties of ILC2s. Here, we collected peripheral blood and liver tissue samples from liver transplant recipients and found that the frequencies of circulating ILC2s were comparable in liver transplant recipients with either rejection or non-rejection compared with the control group, which is consistent with previous findings. Nevertheless, compared with healthy controls, hepatic ILC2s increased dramatically in liver transplant recipients, especially those developing acute rejection. Multiplex immunofluorescence assay revealed significantly increased allograft infiltrating ILC2s in liver transplant recipients, and hepatic ILC2s counts were significantly higher in the rejection group than in the non-rejection group. Similar results were observed for Treg cells. More importantly, we found a close correlation between hepatic ILC2s and Treg cells by analyzing their spatial distribution and distance. Thus, in contrast to the results of the previous study, our study indicated the potential involvement of ILC2s in remodeling the immune microenvironment after LT.

To better understand the impact of ILC2s on the immune status of transplanted liver allografts, we dynamically monitored the changes of ILC2s and their signature cytokines using a mouse orthotopic liver transplant model. We observed that the plasma level of IL-33 and the proportion of ILC2s in liver allografts increased significantly during the early post-transplant period, reaching maximum levels 14 days after LT. It has been reported that IL-33 treatment could significantly prolong islet allograft survival by inducing a long-term expansion of ILC2s, which were required to migrate into the islet allograft to exert maximum graft protection (19). Meanwhile, the proportion of ILC2s in the bone marrow increased significantly after LT, reached maximum levels at seven days after LT, and decreased significantly at 14 days after LT. In contrast, the proportion of ILC2s in the spleen increased dramatically within 14 days after LT. Thus, we presume that the high postoperative plasma level of IL-33 promotes the expansion of ILC2s in bone marrow and spleen and subsequent migration from peripheral blood to liver allograft to exert their immunosuppressive function.

Numerous experimental and clinical studies have indicated that as an immune cell capable of regulating immunity, CD4^+^CD25^+^Foxp3^+^ Treg cells play a crucial role in the induction and maintenance of immune tolerance after LT (22–24). A previous study has demonstrated that migration from blood to the inflamed allograft is required for Treg cells to execute their suppressive function of alloimmunity (25). Likewise, multiplex immunofluorescent assay indicated a significantly increased proportion of graft infiltrating Treg cells in the rejection group compared with the non-rejection and normal groups. More importantly, we firstly found that there was a close correlation between allograft infiltrating ILC2s and Treg cells by analyzing their spatial distribution and distance. Collectively, we speculate that ILC2s may have an immunoregulatory role in posttransplant immune homeostasis akin to Treg cells. However, the precise mechanism by which ILC2s interact with Treg cells *in vivo* remains undetermined.

The main limitation of the present study was the small number of patients and clinical specimen, which makes it difficult to establish conclusions, but the results clearly showed the potential value of the ILC2s in the acute rejection after LT and encouraged continued research with higher numbers of specimens regarding their validation in the clinical process. This study was a single-center study, and thus large multicenter trials are warranted. Furthermore, the limited number and relatively short follow-up of the mouse liver transplant recipients may restrict our study of the changing pattern of ILC2s during the development of spontaneous immune tolerance in liver transplanted mice. Nevertheless, to our knowledge, our study is the first to investigate the allograft infiltrating ILC2s in human and mouse liver transplant recipients.

In summary, the results of the present study suggested that allograft infiltrating ILC2s were dramatically increased in liver transplant recipients, especially those developing acute rejection. We found that there was a close correlation between allograft infiltrating ILC2s and Treg cells by analyzing their spatial distribution and distance. Collectively, we postulate that allograft infiltrating ILC2s may play an inhibitory role in posttransplant immune homeostasis, favoring resolution of liver allograft rejection by interacting with Treg cells or promoting the migration of Tregs cells into the liver allograft. However, the mechanism by which ILC2s interact with Treg cells *in vivo* warrant further research.

## Materials and methods

### Study population

Consecutive liver transplant recipients were enrolled in this study between January 2021 and June 2021 at Beijing Friendship Hospital, Capital Medical University. Patients were included if they (1) received LT for more than one year and (2) underwent liver biopsy for surveillance biopsies or suspected acute rejection. Patients were excluded if they (1) underwent chemotherapy (2), took targeted agents (3), took immunosuppressive drugs other than tacrolimus, mycophenolate mofetil, or steroids (4); had any kind of infections (5), were diagnosed with post-transplant lymphoproliferative disorder (6), received combined organ transplantation, or (7) were diagnosed with chronic rejection. The healthy controls were living liver donors. The clinical and demographic data are shown in **Table 1**. All participants or their guardians provided informed consent to participate in this study. This study was approved by the Ethics Committee of Beijing Friendship Hospital, Capital Medical University. The authors declare that this study adhered to the Declaration of Helsinki.

**Table 1:** Demographic and clinical characteristics of the participants.

Characteristics	Rejection (n=18)	Non-Rejection (n=39)	Healthy control (n=28)	*P-*value
Gender, n (%)				0.7470
Male	12 (66.7%)	22 (56.4%)	16 (57.1%)	
Female	6 (33.3%)	17 (43.6%)	12 (42.9%)	
Age at transplant, year	5.9 (1.4-39.9)	29.7 (1.3-46.3)		0.6552
ALT, U/L	43 (22.5-236)	17 (12.5-27)	14.5 (9-24.5)	< 0.0001
AST, U/L	53.8 (32.9-110.5)	25.6 (19.5-37.1)	19.8 (15.7-22.8)	< 0.0001
TB, μmol/L	14.0 (8.6-20.2)	12.4 (9.6-18.5)	12.1 (10.4-16.7)	0.9243
Original disease, n (%)				0.1150
Biliary atresia	7 (38.9%)	14 (35.9%)		
HBV induced cirrhosis	/	10 (25.6%)		
Inherited metabolic disease	5 (27.8%)	6 (15.4%)		
HBV induced HCC	1 (5.6%)	4 (10.3%)		
Autoimmune cirrhosis	1 (5.6%)	2 (5.1%)		
Alcoholic cirrhosis	2 (11.1%)	/		
Others	2 (11.1%)	3 (7.7%)		
Donor organ type, n (%)				0.5090
Deceased donor	8 (44.4%)	21 (53.8%)		
Living donor	10 (55.6%)	18 (46.2%)		
Cold ischemia time, hours	7.4 (2.1-9.8)	3.1 (1.9-10.7)		0.6921
GRWR, %	1.9 (1.8-2.5)	2.4 (1.7-3.0)		0.5848
Postoperative time, years	3.8 (2.7-6.2)	4.0 (2.9-6.2)		0.5802
Maintenance therapy, n (%)				0.7490
Tacrolimus	10 (55.6%)	23 (60.0%)		
Tacrolimus + MMF	3 (16.7%)	9 (23.1%)		
Tacrolimus + steroids	3 (16.7%)	3 (7.7%)		
Tacrolimus + MMF + steroids	2 (11.1%)	4 (10.3%)		
FK concentration, ng/ml	4.2 (3.0-5.9)	4.2 (2.8-5.7)		0.4875

ALT, alanine aminotransferase; AST, aspartate aminotransferase; GRWR, graft recipient weight ratio; HBV, hepatitis B virus; HCC, hepatocellular carcinoma; HCV, hepatitis C virus; MMF, mycophenolate mofetil; TB, total bilirubin. Categorical variables are described as counts and percentages. Continuous variables are expressed as medians with interquartile ranges.

### Animals

Male wild-type C57BL/6 mice and C3H mice (6-8 weeks old) were purchased from SiPeiFu (Beijing) Biotechnology Co., LTD [license SYXK (Beijing) 2017-0010]. All animals were kept under a specific pathogen-free condition with free access to water and food. All animal experiments in this study were performed in an SPF environment following the ethical guidelines for laboratory animals and were approved by the Institutional Animal Care and Ethics Committee of Beijing Friendship Hospital, Capital Medical University (21–2012).

### Mouse orthotopic liver transplantation model

Wild-type C57BL/6 mice weighing 24-26 g were used as donors, and wild-type C3H mice weighing 26-28 g were used as recipients. All mouse orthotopic LTs were performed by one surgeon who is well-practiced in performing mouse transplantation using a modified “two-cuff” technique approach as described previously (26). After the postoperative recovery of body temperature and rehydration, the recipient mice were kept in the independent ventilation cage facility for further housing. To harvest blood, liver, BM, spleen, and mLN for analysis, the recipient mice were euthanized on day 7 (*n* = 5), day 10 (*n* = 3), and day 14 (*n* = 3) after LT, respectively. Mice in the control group were subjected to a midline incision procedure (*n* = 4).

### Human PBMC isolation and plasma collection

Heparinized blood samples from liver transplant recipients were collected at admission. The blood samples were centrifuged at 1800 rpm for 10 min at room temperature to separate plasma and blood cells. After transferring the supernatant, peripheral blood mononuclear cells (PBMCs) were obtained by density gradient centrifugation using the Lymphocyte Separation Medium (Cat: 7111011, DAKEWE). The plasma and PBMC were stored at -80°C until further use.

### Isolation of immune cells from mice

The liver transplant recipient mice were euthanized by carbon dioxide 7, 10, and 14 days after LT, and the liver, BM, spleen, and mLN were collected for immune cell isolation. The liver was placed on a petri dish containing ice-cold PBS and gently ground through a 70 μm filter. The cell suspension was filtered through a 70 μm cell strainer and then centrifuged at 50g for 5 minutes to obtain the supernatants, purified by 30% Percoll density gradient centrifugation. The lymphocytes in the BM, spleen, and mLN were obtained following methods as previously described (27).

### Flow cytometry

Frozen PBMCs or immune cells were thawed and stained with fluorochrome-conjugated antibodies to surface markers in the dark for 30 minutes at 4°C. To distinguish dead cells, PBMCs or immune cells were stained with LIVE/DEAD Fixable Dead Cell Stain Kits (L34965, Invitrogen) for 30 minutes at room temperature. To stain with antibodies for intracellular markers, including Foxp3 (Foxp3/Transcription Factor Staining Buffer Set; eBioscience), surface stained PBMCs or immune cells were fixed and permeabilized according to the manufacturer’s instructions. The antibodies used are listed in **Supplementary Table 1**. The gating strategies for humans were as follows: CD3+CD4+CD25+CD127^low^ for Treg cells, CD45+Lin-CD127+ for hILC, and on that basis, CRTH2-CD117- for ILC1s, CRTH2+ for ILC2s, CRTH2-CD117+ for ILC3s. The gating strategies for mice were as follows: CD45+Lin-CD90.2+ST2+ for ILC2s, and CD45+CD3+CD4+CD25+Foxp3+ for Treg cells. After being washed with phosphate buffered saline, the cells were analyzed on an Attune flow cytometer (Thermo Fisher), and data analysis was performed using FlowJo software (FlowJo LLC, version 10.0).

### Histology and immunohistochemistry

Liver tissue samples were fixed in 4% neutral buffered formalin, dehydrated, embedded in paraffin, and used for histopathological analysis. 4-μm liver sections were cut and stained with H&E for analysis. The Banff rejection activity index (RAI) score was used for the grading of acute liver allograft rejection (28). We defined the rejection group as RAI ≥ 3 and the non-rejection group as RAI < 3. An experienced liver pathologist performed histological examination and grading of RAI. For immunohistochemistry, allograft infiltrating CD4+ T cells and CD8+ T cells were marked by anti-human CD4 (1:200, ab133616; Abcam), anti-human CD8 (1:200, ab237709; Abcam), anti-mouse CD4 (1:200, ab245118; Abcam), and anti-mouse CD8 (1:200, ab133616; Abcam), separately.

### Multiplex immunofluorescence staining

As previously described, *in situ* multiplex immunofluorescent staining was performed using the Opal Polaris 6 color IHC staining kit (Akoya Biosciences) (29, 30). Briefly, 4 μm thick paraffin-embedded human liver tissue sections were baked for 2 hours at 60°C and then deparaffinized in xylene and rehydrated with a series of graded ethanol solutions. Antigen retrieval was performed using microwave treatment for 20 minutes in antigen retrieval solution pH6 (AR6). After the serial incubation with the following primary antibodies, including anti-CD4, -CD8, -Foxp3, -CD127, and -GATA3 for 1 hour at room temperature, sections were labeled with the anti-rabbit/mouse Polymeric Horseradish Peroxidase (Opal IHC Detection Kit, Akoya Biosciences) for 10 minutes at room temperature. Subsequently, tissue sections were incubated with TSA-conjugated fluorophores (Opal 780 for CD4, Opal 620 for CD8, Opal 480 for Foxp3, Opal 520 for CD127, and Opal 570 for GATA3; PerkinElmer) for 10 minutes. The signal for antibody was visualized by their corresponding Opal Fluorophore (Akoya Biosciences) after a 10-minutes incubation. And a heat-mediated stripping step was inserted between each antibody staining round. Finally, all slides were counterstained with DAPI for 5 minutes, then mounted with an anti-fade mounting medium (P36965, Life Technologies) and stored at four °C before imaging. Image acquisitions (200× magnification as multispectral images) were performed using the Vectra Polaris multispectral imaging platform (Akoya Biosciences), with the entire slide image being scanned and 5–7 representative regions of interest chosen by the pathologist.

Image analysis was performed using the InForm 2.4.8 Image Analysis Software (Akoya Biosciences). Every individual ILC2 or Treg cell in the liver section were identified based on their labeling (CD127+GATA3+ for ILC2; CD4+Foxp3+ for Treg cell), and then the spatial distribution of ILC2s and Treg cells was determined. The distance between ILC2 and the nearest Treg cell and Treg cell and the nearest ILC2 was measured, and then the median distance between ILC2 and Treg cell was calculated. To further clarify the spatial correlation between ILC2s and Treg cells, we counted the number of Treg cells or ILC2s within a circle with a diameter of 15 microns or 25 microns centered on ILC2s and Treg cells, respectively. We drew heatmaps through the R software to exhibit the results.

### Enzyme-linked immunosorbent assay

The cytokine levels of IL-2, IL-4, IL-5, IL-10, IL-13, IL-17, and IL-33 were assayed by ELISA according to the instruction of the manufacturer (all ELISA kits from R&D Systems, Tianjin, China). All samples were measured in duplicate.

### Statistical analysis

Categorical variables are described as counts and percentages. Continuous variables are expressed as medians with interquartile ranges. Comparisons among the three groups were performed by one-way analysis of variance (ANOVA) or Kruskal-Wallis test with *post hoc* Tukey test for pairwise comparison of subgroups. Linear regression analysis was used to determine the correlation between the percent of circulating ILC2s and ALT, AST, and TB. The analyses were conducted using GraphPad Prism version 8.0 (GraphPad Software). A two-sided *p* < 0.05 indicated a significant difference.

## Data availability statement

The raw data supporting the conclusions of this article will be made available by the authors, without undue reservation.

## Ethics statement

The studies involving human participants were reviewed and approved by the ethical committee of Beijing Friendship Hospital, Capital Medical University. Written informed consent to participate in this study was provided by the participants’ legal guardian/next of kin. The animal study was reviewed and approved by the Institutional Animal Care and Ethics Committee of Beijing Friendship Hospital, Capital Medical University (21–2012).

## Author contributions

Z-JZ and L-YS participated in the research design. JS, G-PZ, S-PL, BC, and H-MZ performed the molecular investigations; JS, G-PZ, X-JC, J-MZ, and Y-ZJ participated in *in vivo* and *in vitro* experiment. JS, G-PZ, and S-PL performed the data management and statistical analyses after discussion with all authors. JS and G-PZ wrote the initial draft of the manuscript. All authors were involved in the critical revision of the manuscript for important intellectual content and approved the final version of the manuscript.

## Funding

The present work was supported by the National Natural Science Foundation of China (81970562) and Beijing Postdoctoral Research Foundation. The funding body had no role in the design of the study and collection, analysis, and interpretation of data, and in writing the manuscript.

## Acknowledgments

The authors especially thank Dr. Wei-Tao Que, Department of Hepatobiliary Surgery, Shanghai General Hospital, Shanghai Jiao Tong University, for his help in establishing the mouse orthotopic liver transplantation model.

## Conflict of interest

The authors declare that the research was conducted in the absence of any commercial or financial relationships that could be construed as a potential conflict of interest.

## Publisher’s note

All claims expressed in this article are solely those of the authors and do not necessarily represent those of their affiliated organizations, or those of the publisher, the editors and the reviewers. Any product that may be evaluated in this article, or claim that may be made by its manufacturer, is not guaranteed or endorsed by the publisher.

## Abbreviations

ALT, alanine aminotransferase; APC, antigen-presenting cell; AST, aspartate aminotransferase; BM, bone marrow; ELISA, enzyme-linked immunosorbent assay; GRWR, graft recipient weight ratio; HBV, hepatitis B virus; HCC, hepatocellular carcinoma; HCV, hepatitis C virus; H&E, hematoxylin and eosin; hILC, helper ILC; IL, interleukin; ILC, innate lymphoid cell; ILC1, group 1 innate lymphoid cell; ILC2, group 2 innate lymphoid cell; ILC3, group 3 innate lymphoid cell; LT, liver transplantation; mLN, mesenteric lymph nodes; MMF, mycophenolate mofetil; PBMC, peripheral blood mononuclear cell; POD, postoperative day; RAI, rejection activity index; TB, total bilirubin; Th2 cell, type 2 T helper cell; Treg cell, regulatory T cell.
