# Supplementary material for: Potential correlation of allograft infiltrating group 2 innate lymphoid cells with acute rejection after liver transplantation
Source: Front Immunol. 2022 Jul 28;13:953240. doi: 10.3389/fimmu.2022.953240 (PMC9367675; doi:10.3389/fimmu.2022.953240)
Supplement: Supplementary file 1 [file Table_1.docx]

**Supplementary table 1. Antibody used in the study**

| **Mouse** | | | | |
| --- | --- | --- | --- | --- |
| **Antibody** | **Clone** | **Fluor** | **Product code** | **Company** |
| **Mouse Hematopoietic Lineage Biotin Panel** | — | Biotin | 88-7774-75 | eBioscience |
| **TCRβ** | H57-597 | Biotin | 109203 | BioLegend |
| **TCRγ/δ** | GL3 | Biotin | 118103 | BioLegend |
| **CD4** | GK1.5 | Biotin | 100404 | BioLegend |
| **CD11c** | N418 | Biotin | 117303 | BioLegend |
| **CD5** | 53-7.3 | Biotin | 100603 | BioLegend |
| **CD8a** | 53-6.7 | Biotin | 100704 | BioLegend |
| **NK1.1** | PK136 | Biotin | 108703 | BioLegend |
| **Streptavidin** | — | PE-Cy7 | 25-4317-82 | eBioscience |
| **Streptavidin** | — | APC | 17-4317-82 | eBioscience |
| **Streptavidin** | — | FITC | 11-4317-87 | eBioscience |
| **LIVE/DEAD™ Fixable Aqua Dead Cell Stain Kit** | — | 405 nm excitation | L34965 | Invitrogen |
| **CD45** | 30-F11 | APC-Cy7 | 103116 | BioLegend |
| **CD25** | PC61.5 | FITC/ PE-Cy7 | MA5-17815/25-0251-81 | eBioscience |
| **CD90.2** | 53-2.1 | PE-Cy7/eFluor450 | 25-0902-81 | eBioscience |
| **CD127** | A7R34 | Brilliant Violet 421 | 135027 | BioLegend |
| **GATA3** | TWAJ | PerCP-eFluor 710 | 46-9966-41 | Invitrogen |
| **IL33R/ST2** | DIH9 | PE/APC | 145304 | BioLegend |
| **CD45** | 30-F11 | FITC | 62307S | CST |
| **CD3** | 17A2 | Percp-Cy5.5 | 100218 | BioLegend |
| **CD4** | RM4-5 | FITC | 96127S | CST |
| **CD8a** | 53-6.7 | AF 700 | 100730 | BioLegend |
| **CD25** | PC61.5 | APC | 36055S | CST |
| **Foxp3** | 3G3 | PE-Cy7 | 65210S | CST |
| **Human** | | | | |
| **Antibody** | **Clone** | **Fluor** | **Product code** | **Company** |
| **Lineage cocktail** | — | APC | 22-7776-72 | eBioscience |
| **CD45** | HI30 | APC-Cy7 | 47-0459-42 | eBioscience |
| **CD127** | EBioRDR5 | eFluor 450 | 48-1278-42 | eBioscience |
| **CRTH2** | BM16 | PE | 12-2949-42 | eBioscience |
| **CD117** | 104D2 | FITC | 11-1178-42 | eBioscience |
| **CD161** | HP-3G10 | PE-Cy7 | 25-1619-42 | eBioscience |
| **IL-13** | JES10-5A2 | PerCP/Cy5.5 | 501912 | Biolegend |
| **CD3** | HIT3a | FITC | 300306 | BioLegend |
| **CD4** | SK3 | AF 700 | 344622 | BioLegend |
| **CD8** | RPA-T8 | BV 605 | 301040 | BioLegend |
| **CD25** | M-A251 | BV 450 | 560355 | Bioscience |
| **CD25** | BC96 | BV421 | 302630 | Biolegend |
| **CD127** | A019D5 | APC | 351316 | Biolegend |
| **Foxp3** | PCH101 | APC | 17-4776-42 | Bioscience |
